# Akt Regulates IL-10 Mediated Suppression of TNFα-Induced Cardiomyocyte Apoptosis by Upregulating Stat3 Phosphorylation

**DOI:** 10.1371/journal.pone.0025009

**Published:** 2011-09-20

**Authors:** Sanjiv Dhingra, Ashim K. Bagchi, Ana L. Ludke, Anita K. Sharma, Pawan K. Singal

**Affiliations:** Department of Physiology, Faculty of Medicine, Institute of Cardiovascular Sciences, St. Boniface General Hospital Research Center, University of Manitoba, Winnipeg, Canada; Emory University, United States of America

## Abstract

**Background:**

We have already reported that TNF-α increases cardiomyocyte apoptosis and IL-10 treatment prevented these effects of TNF-α. Present study investigates the role of Akt and Jak/Stat pathway in the IL-10 modulation of TNF-α induced cardiomyocyte apoptosis.

**Methodology/Principal findings:**

Cardiomyocytes isolated from adult Sprague Dawley rats were exposed to TNF-α (10 ng/ml), IL-10 (10 ng/ml) and TNF-α+IL-10 (ratio 1) for 4 h. Exposure to TNF-α resulted in an increase in cardiomyocyte apoptosis as measured by flow cytometry and TUNEL assay. IL-10 by itself had no effect, but it prevented TNF-α induced apoptosis. IL-10 treatment increased Akt levels within cardiomyocytes and this change was associated with an increase in Jak1 and Stat3 phosphorylation. Pre-exposure of cells to Akt inhibitor prevented IL-10 induced Stat3 phosphorylation. Furthermore, in the presence of Akt or Stat3 inhibitor, IL-10 treatment was unable to block TNF-α induced cardiomyocyte apoptosis.

**Conclusion:**

It is suggested that IL-10 modulation of TNF-α induced cardiomyocyte apoptosis is mediated by Akt via Stat3 activation.

## Introduction

Proinflammatory cytokine tumor necrosis factor-α (TNF-α), has been shown to have a cardiodepressent effect and is involved in various cardiovascular complications [1], [2]. Sustained long-term over-expression of TNF-α provokes the induction of cardiomyocyte apoptosis [3], which contributes to the pathophysiology of several heart diseases, including dilated cardiomyopathy, myocardial infarction, and heart failure [4], [5]. Surprisingly, anti-TNF-α therapy had no benefit or even had harmful effects in patients with heart failure [6], [7]. Thus identification of the intermediary steps involved in the TNF-α induced cardiomyocyte apoptosis can offer potential therapeutic targets to abrogate TNF-α induced cardiovascular diseases.

The anti-inflammatory cytokine interleukin-10 [IL-10], inhibits the production of various pro-inflammatory cytokines including TNF-α [8]. After binding to its receptors IL-10R1 or IL-10R2 on the cell surface [9], IL-10 activates Jak/Stat pathway [10]. It has also been reported that IL-10 activates ERK1/2 by inducing tyrosine phosphorylation, therefore supporting cell survival and cell protection [11].We have recently reported that TNF-α increases cardiomyocyte apoptosis by activating p38 MAP kinase and NFκB pathway and by down-regulating ERK 1/2 MAP kinase [12], [13]. IL-10 treatment prevented these effects of TNF-α in isolated cardiomyocytes and we also found increased activation of ERK1/2 MAPK upon IL-10 treatment [12]. However the precise site of IL-10 action in this process is still unknown.

Akt, a serine-threonine kinase, regulates cellular metabolism, is pro-survival and proliferative/growth effects. Among numerous signaling pathways involved in the regulation of cellular apoptosis, Akt plays a crucial role [14], [15]. It is activated downstream of phosphatidylinositol 3-kinase (PI3K) in response to stimulation of receptor tyrosine kinases [16], [17]. After activation, Akt is thought to regulate pro-survival gene transcription through different pathways [18], [19]. It has been shown that IL-10 promotes the survival of astrocytes by a mechanism that involves an activation of PI 3-kinase [20]. In another report, it has been shown that IL-10 promotes survival of myeloid progenitors by activating Akt pathway through the involvement of ERK 1/2 [21] and tyrosine kinase [11]. Hence, in continuation to our recent study regarding the role of ERK1/2 in IL-10 modulation of TNF-α induced effects in cardiomyocyes, here we have investigated further downstream signalling mechanisms involved in cardiomyocyte survival process. Present study indicates that IL-10 modulates TNF-α induced cardiomyocyte apoptosis by upregulating Akt via Stat3 phosphorylation.

## Materials and Methods

The investigation conforms to the Guide for the Care and Use of Laboratory Animals published by the US National Institutes of Health (NIH Publication No. 85-23, revised 1996). All animal-experiment protocols were approved by the University of Manitoba Animal Care Committee following the guidelines established by the Canadian Council on Animal Care.

### Isolation of adult ventricular cardiomyocytes

Cardiomyocytes were isolated from normal adult male Sprague Dawley (SD) rats (250–300 g) using modified Langendorff perfusion apparatus as described previously [22]. Cardiomyocytes (1×10^6^ per well) were plated on laminin- coated polystyrene tissue culture dishes. Plated cells were incubated in serum-free culture medium M199 supplemented with antibiotics (streptomycin/penicillin, 100 µg/ml) at 37°C under a 5% CO_2_-95*%* air atmosphere. Two hours after plating, the culture medium was changed to remove unattached dead cells and the viable cardiomyocytes were incubated overnight under the same culture conditions.

### Treatment with cytokines

After initial incubation of 24 h, more than 90% of cardiomyocytes were viable and these cells were treated with one of the following; TNF-α (10 ng/ml), IL-10 (10 ng/ml), or combination of TNF-α+IL-10 (ratio 1) and H_2_O_2_ (100 µM) for 4 h. Cardiomyocytes were treated with 20 µmol/L of trolox for 30 min and then incubated with the combination of trolox and TNF-α (10 ng/ml) for 4 h. Hydrogen peroxide (H_2_O_2_) and trolox were used as positive controls to mimic oxidative stress and antioxidant, respectively [12], [13]. In order to study the role of Akt and Stat 3 in the IL-10 regulation of TNF-α induced cardiomyocyte apoptosis, cells were pretreated with 50 µM of LY294002 (PI3K/Akt inhibitor) or 50 µM of ‘5, 15 DPP’ (Stat 3 inhibitor) or with DMSO (vehicle solution, at a final concentration of 0.01%) for 30 min. These concentrations of inhibitors are based on our pilot studies as well as manufacturer's data sheet for IC 50 for each inhibitor. After each inhibitor treatment, cells were washed three times to remove any residual inhibitor that can interfere with TNF-α or IL-10 treatment. The concentrations and time of exposures for TNF-α and IL-10 are based on our earlier studies [12], [22].

### Cell viability and apoptosis

#### Cell viability

Trypan blue exclusion method was used to study the viability of the cardiomyocytes. Briefly, 0.4% trypan blue was added directly to the cells suspended in PBS. Cells were counted and expressed as percentage of viable cells.

#### Flow cytometry

The Vybrant® Apoptosis assay kit # 7 (Molecular Probes, V-23201) was used to detect cardiomyocyte apoptosis by flow cytometry (FACS Calibur, Becton Dickinson). After different treatments, cardiomyocytes were washed with PBS and adjusted the cell density to 1×10^6^ cells/ml in PBS. One microlitre of YO-PRO-1 and propidium iodide (PI) was added according to manufacturer's guide. The mixture was incubated on ice in the dark for 20 min. After staining with YO-PRO-1 and PI, apoptotic cells show green fluorescence and dead cells show red fluorescence. The fluorescence was measured by flow cytometry at emission wavelengths ∼530 and ∼575 nm for the detection of YO-PRO-1 and PI, respectively. Dot plot analysis was done for 20,000 events using Cell Quest Software (Bector Dickinson).

#### Terminal Deoxyribonucleotidytransferase mediated deoxyuridine triphosphate nick end labelling (TUNEL assay)

Cardiomyocyte apoptosis was also studied by TUNEL assay as described previously [23], [24]. Cells (10^5^ cells/ml) from different treatment groups were cultured in 8-well chamber slide and fixed in 4% paraformaldehyde followed by digestion with proteinase K (10 µg/ml) for 15 min at 37°C and permiabilization with 0.1% triton X-100 for 5 min at 4°C. After washing twice with PBS, cardiomyocytes were incubated with a mixture of 1 µl of Terminal Deoxynucleotidyltransferase (TdT) enzyme and 4 µl of fluorescein isothiocyanate (FITC) labelled 12dUTP in equilibrium buffer (Genescript, USA). Cells were then assayed under fluorescence microscope using excitation wavelength 450–500 nm and emission wavelength 515–560 nm. Finally, TUNEL positive cells were counted.

### Western blot analysis

Whole-cell protein extracts prepared from control and treated cardiomyocytes in different groups were suspended in PBS containing protease inhibitor cocktail. Electrophoresis, immunoblotting, and protein detection were done for Jak1, Stat3, Akt, Bax and Bcl-xl using specific antibody kits (Cell Signaling Technology, USA) as described previously [13]. Bands were visualized with Fluor S-MultiImager MAX system (Bio-Rad Laboratories, Canada) and quantified by image analysis software (Quantity One, Bio-Rad Laboratories, Canada).

### Cell-Based ELISA for Akt phosphorylation

Phosphorylation of Akt (Ser473) was detected by using fluorogenic substrates provided in the ELISA kit (R&D Systems) according to manufacturer's protocol. Briefly, cells were plated and treated in the 96 well microplate, then fixed with 4% paraformaldehyde in PBS. After blocking, cells were treated with primary and then secondary antibodies. After incubation with the substrate, fluorescence intensity in the plate was read using plate reader with excitation at 540 nm and emission at 600 nm for phosphorylated Akt in the cells. For total Akt, the plates were read with excitation at 360 nm and emission at 450 nm.

### Caspase-3 activity assay

Caspase-3 activity was measured by spectrofluorometric assay using the fluoro-chrome 7-amino-4-methyl coumarin (AMC) and substrate DEVD-AMC, provided in the CaspACE™ assay system (Promega Corp. Madison, USA). The assay was performed in 96 well polystyrene plates (Becton Dickinson, USA). From each treatment group 100 µg of protein was added to the reaction wells, together with 32 µl of caspase assay buffer, 2 µl DMSO, 10 µl of dithiothreitol and 2 µl of DEVD-AMC substrate (dissolved in DMSO). The reaction mixture was incubated at 30°C for 60 minutes and thereafter analyzed for AMC fluorescence using spectrofluorometer (Spectra Max Gemini, USA) at 360 nm excitation and 460 nm emission. The fluorescence data were corrected by subtracting background values.

### Immunofluorescence

Cardiomyocytes after different treatments were fixed with 4% paraformaldehyde. For localization of Akt, cells were stained overnight with rat anti phospho-Akt antibody (Cell Signalling Technology, USA; 1∶40) and then secondary antibody Alexa Fluor® 555 (Invitrogen Inc., USA; 1∶250) for 3 hours. Simultaneously, for actin filaments, cells were stained with fluorescein phalloidin (Molecular Probes). After secondary labelling cells were washed with PBS and fixed with ProLong® Gold antifade reagent. The fluorescence signals were examined using Fluorescence microscope (Olympus, Japan).

### Protein and Statistical analysis

Total protein concentration was determined using bovine serum albumin as a standard [25]. Data are expressed as the mean ± SEM. Groups were compared by one-way ANOVA and Bonferroni's test was performed to identify differences between groups. Value of p<0.05 was considered significant.

## Results

### Cardiomyocyte apoptosis and viability

**Figure 1:**
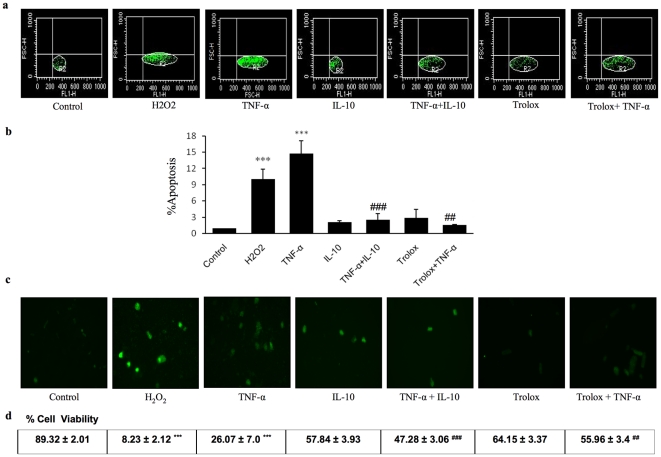
Effects of H_2_O_2_ (100 µM), TNF-α (10 ng/ml), IL-10 (10 ng/ml), TNF-α+IL-10 (ratio 1), Trolox (20 µmol/L) and Trolox+TNF-α on apoptosis and cell viability in adult rat cardiomyocytes. **a**) Representative dot plots of flow cytometric images of cardiomyocytes; **b**) Quantitative analysis of apoptosis. Data are mean ± SEM from 4 different experiments; c) Representative fluorescence microscopic images of cardiomyocytes after TUNEL assay; and **d**) Percentage of viable cariomyocytes as assessed by trypan blue assay. *** Indicates significantly different (p<0.001) from the respective control group; Significantly different (### p<0.001; ## P<0.01) when compared with TNF-α group.

Cardiomyocyte apoptosis and viability were studied by flow cytometry and TUNEL assay (Figs. 1a–d). Exposure to TNF-α resulted in an increase in apoptosis. Treatment with IL-10 alone did not show any significant change, whereas TNF-α induced increase in the number of apoptotic cells was significantly prevented by IL-10 treatment. H_2_O_2_ treatment, used as a positive control for oxidative stress, also increased the level of apoptotic cells as compared to untreated cells. Antioxidant trolox significantly prevented the TNF-α-induced cardiomyocyte apoptosis. Trolox by itself had no effect.

TNF-α exposure to cardiomyocytes significantly decreased the cell viability. IL-10 treatment prevented TNF-α induced decrease in cell viability. H_2_O_2_ also led to a significant decrease in cardiomyocyte viability and antioxidant trolox prevented the TNF-α-induced decrease in cardiomyocyte viability (Fig. 1d).

### Effects of TNF-α and IL-10 on Akt phosphorylation

**Figure 2:**
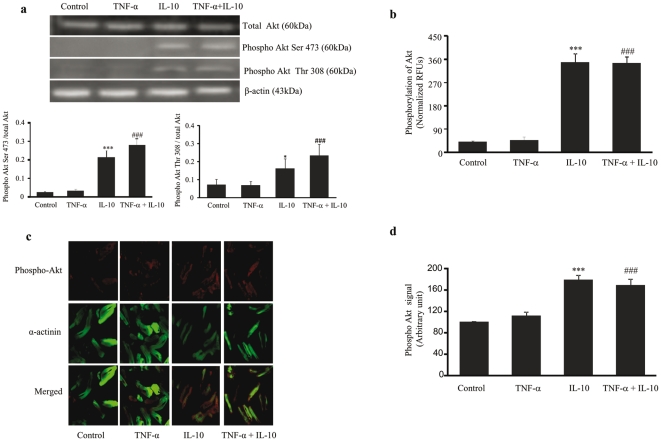
Effects of TNF-α (10 ng/ml), IL-10 (10 ng/ml), TNF-α+IL-10 (ratio 1) on Akt phosphorylation in adult rat cardiomyocytes. **a**) **Upper panel**, western blot analysis using specific Akt antibodies and β-actin as an internal control; **Lower panel**, densitometric analysis and the values are ratios of phosphorylated to total Akt serine 473 (left panel) and threonine 308 (right panel); and **b**) Show Akt phosphorylation as measured by cell based ELISA, values are normalized as relative fluorescence units (RFU's). **c and d**) Immunofluorescence, cells were stained with phospho-Akt antibody and secondary antibody Alexa Flour 555. Flourescein phalloidin was used to stain α-actinin. Representative fluorescence microscopic images of cardiomyocytes (**c**). Quantification of the Akt expression. Data are expressed as mean±SEM from 4–6 different experiments (**d**). *p<0.01 and ***P<0.001 significantly different from the control group; ###p<0.001 significantly different when compared with TNF-α group.

Akt, a serine/threonine kinase, once activated within the cell, regulates the transcription of several pro-survival genes. We examined the phosphorylation of Akt in the cardiomyocytes exposed to TNF-α (10 ng/ml), IL-10 (10 ng/ml) and IL-10+TNF-α (ratio 1) at serine 473 and threonine 308 sites. Exposure to TNF-α did not cause any change in Akt phosphorylation as measured by Western blot and cell-based ELISA (Figs. 2a and b). IL-10 treatment significantly (p<0.001) increased Akt phosphorylation at both serine 473 (Fig. 2a, lower left panel) and threonine 308 sites (Fig. 2a, lower right panel) and this positive effect of IL-10 was also seen in the presence of TNF-α. Immunofluorescence measurements (Figs. 2c and d) complemented the above findings seen with Western blot and ELISA (Figs. 2a and b).

### Inhibition of Akt pathway

**Figure 3:**
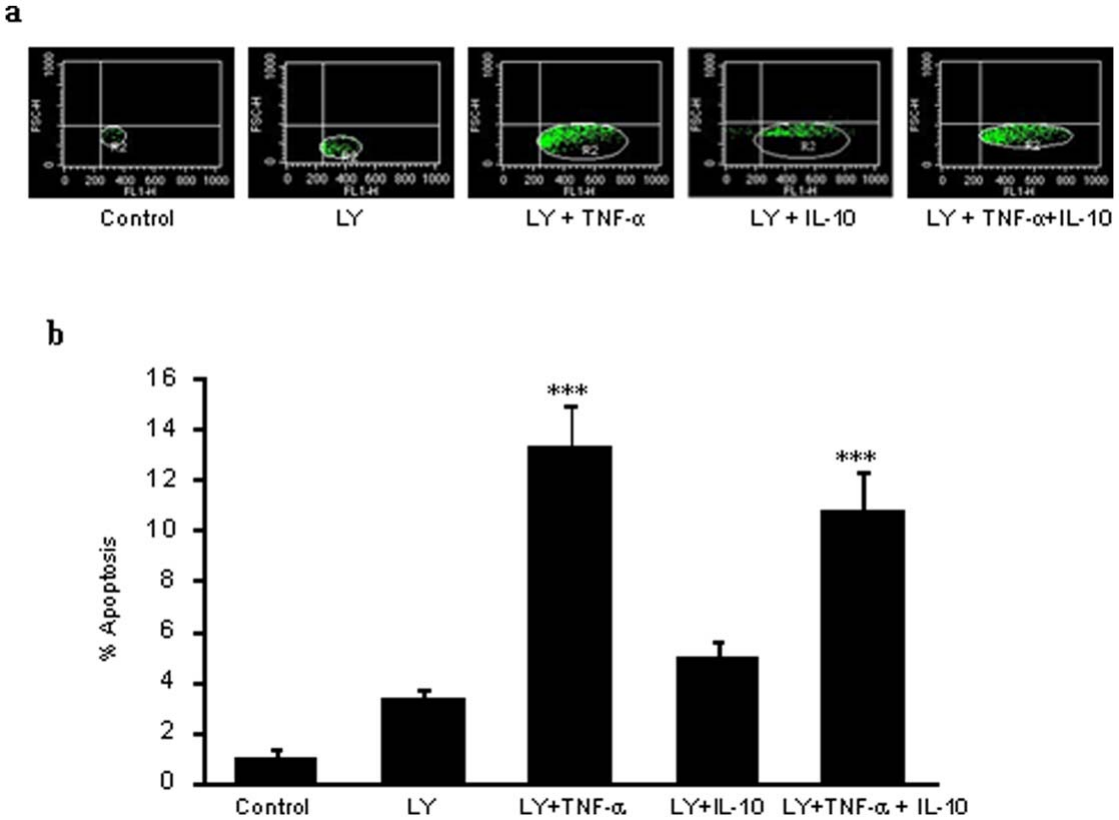
Effects of TNF-α (10 ng/ml), IL-10 (10 ng/ml) and TNF-α+IL-10 (ratio 1) on apoptosis in adult rat cardiomyocytes after Akt inhibition. Cells were pretreated with 50 µM of PI3K/AKT inhibitor LY294002 (LY) for 30 minutes. **a**) Representative dot plots of flow cytometric images of 20,000 events. **b**) Quantification of the level of apoptosis using YO-PRO. Data are mean ± SEM from 4 different experiments. Significantly different (***p<0.001) from the control group containing 0.01% DMSO.

In order to study the role of Akt pathway in the IL-10 regulation of TNF-α induced cardiomyocyte apoptosis, we pre-exposed cardiomyocytes to PI3K/Akt inhibitor (LY294002) and then treated the cells with TNF-α and IL-10 and checked the level of apoptosis. In Akt inhibited cells, IL-10 treatment failed to block the TNF-α induced cardiomyocyte apoptosis (Figs. 3a and b) as seen in presence of TNF-α+IL10 (Fig. 1b). In studies employing different inhibitors dissolved in DMSO (0.01%), the controls were also supplemented with this vehicle.

**Figure 4:**
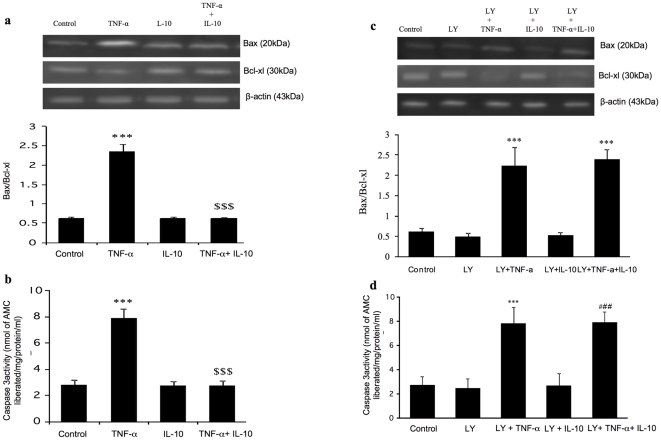
Effects of TNF-α (10 ng/ml), IL-10 (10 ng/ml), and TNF-α+IL-10 (ratio 1). on Bax/Bcl-xl ratio and caspase-3 activity in adult rat cardiomyocytes (a & b). **a**) Bax/Bcl-xl data, **Upper panel:** Western blot analysis using specific Bax, Bcl-xl antibody and β-actin was used as an internal control; **Lower panel:** Shows densitometric analysis of Bax/Bcl-xl. **b**) Caspase-3 activity as measured in terms of flouro-chrome, amino methyl coumarin (AMC), liberated in the fluorometric assay system (CaspACE™). Bax/Bcl-xl ratio and caspase-3 activity after Akt inhibition (**c & d**). Cells were pretreated with 50 µM of PI3K/AKT inhibitor LY294002 (LY) for 30 minutes. **c**) **Upper panel:** Western blot analysis using specific Bax, Bcl-xl antibody and β-actin was used as an internal control; **Lower panel:** Shows densitometric analysis. **d**) Caspase-3 activity. Data are expressed as mean±SEM from 4–6 different experiments. Significantly different (***p<0.001) from control; $$$p<0.001 vs respective TNF-α group; ###p<0.001 vs respective LY+IL-10 group. In figure 4c and d, the control had 0.01% DMSO.

TNF-α caused an increase in the ratio of Bax/Bcl-xl and caspase-3 activation and IL-10 treatment of cardiomyocytes blocked this effect of TNF- α (Figs. 4a and b). Furthermore, we observed that in cardiomyocytes pre-treated with Akt inhibitor, TNF-α caused an increase in both Bax/Bcl-xl ratio and caspase activity and IL-10 was not able to prevent these TNF-α induced changes (Figs. 4c and d). To confirm whether PI3K inhibitor, LY294002, actually inhibited Akt phosphorylation, cells were pre-incubated with 50 µM of LY294002 for 30 min and Akt expression was measured. Treatment with LY294002 inhibited IL-10 induced Akt phosphorylation (data not shown).

### Involvement of Jak/Stat pathway

**Figure 5:**
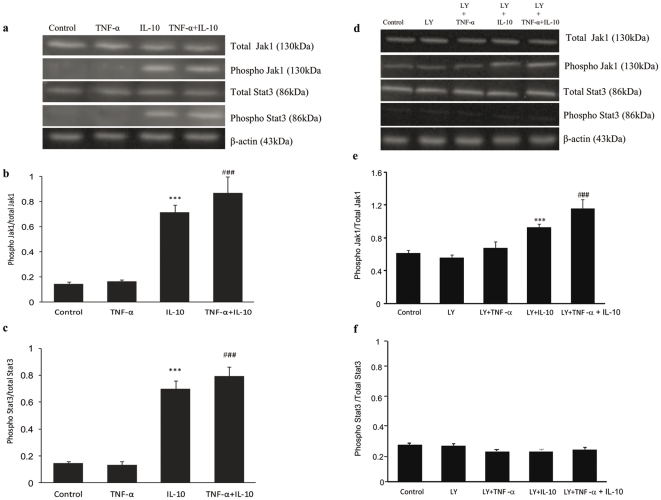
Jak1 and Stat3 phosphorylation in adult rat cardiomyocytes after treatment with TNF-α (10 ng/ml), IL-10 (10 ng/ml) and TNF-α+IL-10 (ratio 1) with or without pretreatment of PI3K/Akt inhibitor Ly294002 (LY) for 30 min. Western blot (**a & d**) and densitometric analysis to calculate the ratio of phosphorylated to total, Jak1 (**b & e**) and Stat3 (**c & f**) expression. The relative levels of protein expression were normalized to β-actin used as an internal control. Data are expressed as mean±SEM from 4–6 different experiments. Significantly different (***p<0.001) from respective control; ###p<0.001 vs. respective TNF-α group. In figure 5d, e and f, the control had 0.01% DMSO.

IL-10 after binding to its receptors on the cell surface activates Jak/Stat pathway that further up-regulates several nuclear transcription factors involved in cell survival. We have investigated whether IL-10 mediated activation of Akt is involved in cell survival through Jak/Stat pathway. We examined the Jak1 and Stat3 phosphorylation in cardiomyocytes exposed to TNF-α (10 ng/ml), IL-10 (10 ng/ml) and TNF-α+IL-10 (ratio 1). Exposure to TNF-α did not cause any change in Jak1 and Stat3 phosphorylation (Figs. 5a, b, and c). While, IL-10 treatment, significantly (p<0.001) increased the Jak1 and Stat3 phosphorylation as compared to control, as well as TNF-α. This IL-10-induced increase in Jak1 and Stat3 phosphorylation was also seen in the presence of TNF-α (P<0.001).

**Figure 6:**
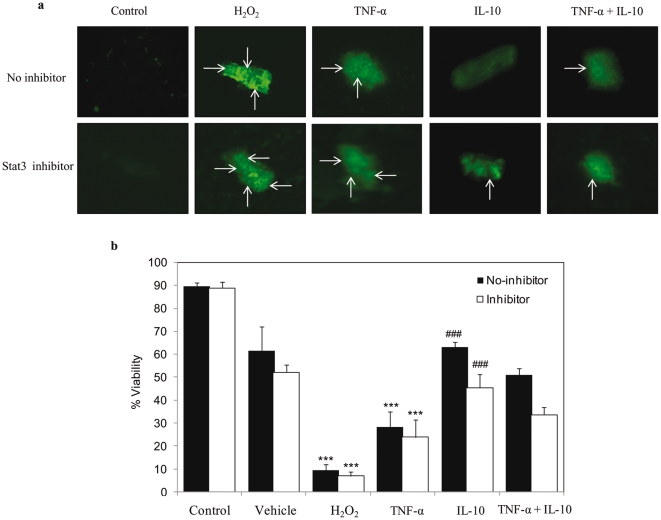
Effects of H_2_O_2_ (100 µM), TNF-α (10 ng/ml), IL-10 (10 ng/ml), and TNF-α+IL-10 (ratio 1), on apoptosis in adult rat cardiomyocytes after Stat3 inhibition. Cells were pretreated with 50 µM of Stat3 inhibitor (5,15DPP) for 30 minutes and for the control cells the medium contained 0.01% DMSO. Fluorescence microscopic images of cardiomyocytes after TUNEL assay are representative from 3 different experiments. Images were taken at 20× magnification and further enlarged to 210×210 pixels. **b**) Percentage of viable cardiomyocytes as assessed by trypan blue assay. ***) Indicates significantly different (p<0.001) from the respective control group; ###) significantly different (p<0.001) as compared with respective IL-10 and TNF-α+IL-10 no-inhibitor group.

In cardiomyocytes pre-treated with Akt inhibitor, we still observed IL-10 induced significant increase in Jak1 phosphorylation which was also seen in the presence of TNF-α (Figs. 5d and e). However, Akt inhibitor did prevent IL-10 induced increase in Stat 3 phosphorylation (Figs. 5d and f). To further investigate the direct role of Stat3 in the interplay of TNF-α and IL-10 in regulating cardiomyocyte apoptosis, the level of apoptosis was checked in the presence of Stat3 inhibitor (5, 15 DPP). We observed that in Stat3 inhibited cells, IL-10 treatment was not able to prevent TNF-α induced cardiomyocyte apoptosis and cell viability (Fig. 6a and b).

## Discussion

Earlier we have reported that TNF-α induces cardiomyocyte apoptosis and this effect is mediated by an increase in oxidative stress [12], [13]. Furthermore, IL-10 was shown to mitigate this effect via its antioxidant ability as well as an increase in ERK1/2 MAPK activity [12], [13]. It is also known that JAK/Stat and PI3K/Akt provide resistance against oxidative stress (caused by H_2_O_2_) as well as apoptosis in neonatal rat cardiomyocytes [26]. It was shown that cells surviving oxidative stress had 2.4 and 2.8 folds higher levels of Akt and Stat3, respectively in neonatal rat cardiomyocytes [26]. The present study examines this downstream signalling mechanism in adult rat cardiomyocytes and the data show that this beneficial effect of IL-10 may involve an upregulation of Akt phosphorylation that activates the transcription factor Stat-3.

Apoptosis plays a key role in the pathogenesis of cardiovascular disease due to loss of terminally differentiated cardiomyocytes. The pro-apoptotic role of TNF-α has been extensively investigated in a variety of *in vitro* and *in vivo* models [12], [13], [14], [27]. TNF-α causes apoptotic cell death by two different but interrelated pathways [28]. First, by binding to its cell surface receptors, TNF-α triggers apoptotic signalling through caspase-3 activation and causes apoptosis (extrinsic pathway). Second, TNF-α can also stimulate apoptosis as a result of overproduction of reactive oxygen species and mitochondrial injury (intrinsic pathway). In this regard, we have previously reported that direct exposure of cardiomyocytes to TNF-α led to an increase in the production of reactive oxidative species and an increase in oxidative stress [12]. Such an increase in oxidative stress disturbs the balance between anti- and pro-apoptotic proteins of Bcl-2 family, thus disrupting the mitochondrial membrane potential and inducing the release of cytochrome c and finally leading to apoptosis. Anti-TNF-α therapies in patients with heart failure were not effective, a precise reason for this is unknown [6], [7], [29].

IL-10, an anti-inflammatory cytokine, inhibits the production of several pro-inflammatory cytokines including TNF-α. An imbalance between IL-10 and TNF-α causes cell injury and an increase in oxidative stress in isolated cardiomyocytes [30], [31]. In an *in-vivo* study in rats, it has been observed that the progression of experimentally induced heart failure subsequent to myocardial infarction was associated with a decrease in IL-10/TNF-α ratio [19], [21]. Patients with advanced congestive heart failure (New York Heart Association Class III and IV) also showed low IL-10 to TNF-α ratio [32]. These observations provide evidence that improved heart function is associated with improved IL-10/TNF-α ratio [22], [30]. TNF-α induced cardiomyocyte apoptosis, in the present study, was significantly decreased by IL-10 treatment. Pro-apoptotic effects of TNF-α have been shown to be modulated by IL-10 treatment in other cell types as well. In this regard, in human articular chondrocytes, IL-10 upregulated bcl-2 expression and down-regulated TNF-α induced caspase-3 activity, thus decreased the level of apoptosis [33]. The intracellular mechanism by which IL-10 generates survival signals and mediates its anti-inflammatory effects remains largely unknown. In this study, we show an involvement of Akt, a serine/threonine kinase, in the protective role of IL-10 in TNF-α induced cardiomyocyte apoptosis.

Akt, also known as protein kinase B, has emerged as a focal point for signal transduction pathways responsible for cell survival, energy metabolism and protein synthesis [14]. In cardiomyocytes, Akt prevents apoptosis due to ischemia reperfusion [16], [34], volume and/or pressure overload and hypoxia [17]. Several drugs, beneficial to heart patients, are known to act through Akt signalling [35]. It has been reported that amlodipine improves cardiac function through the activation of Akt pathway and by inhibiting the level of TNF-α [35]. In the present study, exposure to TNF-α did not cause any significant change in Akt phosphorylation. However, IL-10 treatment by itself caused a significant increase in Akt phosphorylation and TNF-α exposure did not affect this IL-10 induced increase. In order to further investigate, whether IL-10 regulates TNF-α induced cardiomyocyte apoptosis through Akt mediated cell survival pathway, we pre-exposed the cells to PI3K/Akt pathway inhibitor (LY-294002) and examined the effects of TNF-α and IL-10 on cardiomyocyte apoptosis. In LY-294002 treated cells, IL-10 treatment was not able to block TNF-α induced caspase-3 activation and cardiomyocyte apoptosis. These data suggest that Akt mediates IL-10 regulation of TNF-α induced cardiomyocyte apoptosis.

Regulation of intracellular apoptotic processes by Akt is mediated through the Bcl-2 family of proteins. It has been reported that leukemia inhibitory factor (LIF) prevented doxorubicin induced cardiomyocyte apoptosis by activating PI3K/Akt that further maintained Bcl-xl level [36]. Another Bcl-2 family protein regulated by Akt is the proapoptotic BH1-3 protein, Bax, which is a key molecule in mitochondrial outer membrane permeabilization [37]. Akt inhibits Bax activation and apoptosis in a PI3K-dependent manner [18]. In the present study, Akt also mediated the IL-10 regulation of TNF-α induced cardiomyocyte apoptosis by preventing an increase in the ratio of Bax and Bcl-xl. Furthermore, in LY-294002 treated cells, IL-10 treatment was not able to prevent TNF-α induced increase in the ratio of Bax/Bcl-xl.

It is now well accepted that IL-10, after binding to its receptors on the cell surface, activates Jak/Stat pathway. In the present study, TNF-α exposure did not cause any change in Jak1 and Stat3 phosphorylation. Whereas, IL-10 treatment significantly increased both Jak1 and Stat3 phosphorylation. When cardiomyocytes were pre-treated with PI3K/Akt inhibitor (LY294002), IL-10 treatment failed to increases Stat3 phosphorylation. In this regard, Stat3 phosphorylation has also been reported to occur through other members of the Janus family of non-receptor tyrosine kinases [35], [36], [37]. We have also observed that Akt inhibition in the present study could not block IL-10 induced increase in Jak1 phosphorylation. Furthermore in the presence of Stat3 inhibitor IL-10 treatment was not able to prevent TNF-α induced apoptosis and cell viability was decreased. Thus, this study provides evidence that TNF-α induced cardiomyocyte apoptosis is mitigated by IL-10 treatment via the up-regulation of Akt phosphorylation that further increases Stat3 phosphorylation independent of JAK1 activation.
